# Children's Country Holidays Fund

**Published:** 1889-09-07

**Authors:** 


### Children's Country Holidays Fund.

On August 21st the Chairman of the London School Board spoke, in a letter to the *Times*, of the value of the Children's Country Holidays Fund in the elementary schools. While drawing attention to the vast numbers of children in the poorest schools, he praised the care and discrimination with which the beneficiaries of the fund were selected, and even added that while hearing much of its usefulness he had heard of no cases in which it had been abused. It is a matter of congratulation that the School Board feels so cordially towards the movement. It might well have been that the board and its teachers would have taken alarm at possible dangers to "attendance." It is, of course, manifest that some children must be sent away during school-time, for to send 20,000 into the country during the three or four weeks during which the school holidays last would require an enormous organisation and large sums of money in hand by the middle of July. The capacity of the 450 villages now used for the little visitors would have to be doubled or trebled, since not many more than 7,000 can apparently be received among them at the same time. The school authorities, however, recognise the efforts made by the committees to minimise interference with school work and school attendance. The teachers see more clearly than anyone the great physical benefit the children derive from their country stay, and are sensible of the mental enlargement which results. The children see with their own eyes illustrations of what they have read in school. We heard the other day of some little girls who rushed to tell their teacher of a real cow "chewing the cud"—an action previously unintelligible, though so often insisted on in their books.

It is, indeed, hardly possible to realise the mental position of the boys and girls who grow up without any acquaintance with the commonest incidents and the daily sights and sounds of country life. So many of their lessons must be unmeaning to those who, if they have ever penetrated beyond London chimney pots, have only done so in their hundreds at some school treat—where tea, and buns, and roundabouts were the real events of the "day in the country."

